# Aspirin or non‐steroidal anti‐inflammatory drug initiation and subsequent bladder cancer evaluation

**DOI:** 10.1111/joim.70115

**Published:** 2026-06-01

**Authors:** Malene Söth Hansen, Lars Dyrskjøt, Uffe Heide‐Jørgensen, Jørgen Bjerggaard Jensen, Mette Nørgaard

**Affiliations:** ^1^ Department of Clinical Epidemiology Aarhus University Hospital Aarhus Denmark; ^2^ Department of Clinical Medicine Aarhus University Aarhus Denmark; ^3^ Department of Molecular Medicine Aarhus University Hospital Aarhus Denmark; ^4^ Department of Urology Aarhus University Hospital Aarhus Denmark

**Keywords:** aspirin, bladder cancer, cancer early diagnosis, cystoscopy, NSAID

## Abstract

**Background:**

Whether aspirin or non‐aspirin non‐steroidal anti‐inflammatory drugs (NSAIDs) initiation may unmask unknown bladder cancers (BCs) by hematuria remains controversial.

**Objectives:**

To evaluate unmasking, we compared cystoscopy rates, BC prevalence at cystoscopy, and invasive stage prevalence at diagnosis in aspirin or NSAID initiators with never‐users.

**Methods:**

Using the Danish population‐based registries, we identified all new aspirin or NSAID users in 2005–2023 and a matched never‐using comparison cohort. Second, we identified all first‐time cystoscopy recipients in 2005–2022 with aspirin or NSAID initiation within 1 year prior or never‐use in 10 years preceding cystoscopy. Comparing these groups, we computed hazard ratios of cystoscopy during 1‐year follow‐up, prevalence ratios of BC at cystoscopy, and invasive stage disease at diagnosis.

**Results:**

Among 50,771 aspirin initiators (median age: 67 years, 51% men) and 156,191 NSAID initiators (age: 45 years, 50% men) adjusted hazard ratios of cystoscopy were 1.54 (95% confidence interval [CI]: 1.38–1.72) and 1.56 (95% CI: 1.43–1.70) compared with never‐users. Cystoscopy recipients included 683 aspirin (age: 70 years, 69% men) and 1414 NSAID initiators (age: 60 years, 63% men); standardized morbidity ratio weighted (SMRW) prevalence ratios were 0.97 (95% CI, 0.76–1.22) and 0.65 (95% CI, 0.51–0.82) compared with never‐users. The SMRW invasive stage prevalence ratios were 0.81 (95% CI, 0.63–1.03) and 1.07 (95% CI, 0.85–1.34) in the aspirin and NSAID comparison.

**Conclusion:**

The combination of more cystoscopies and a lower prevalence of invasive stage at BC diagnosis may suggest the unmasking of asymptomatic BC in aspirin initiators. We did not find indications of unmasking with NSAID initiation.

## Graphical abstract

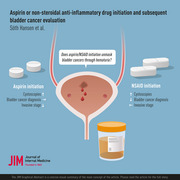

**Abbreviations**
NSAID: non‐aspirin, non‐steroidal, anti‐inflammatory drug
COX: cyclooxygenase enzyme
CCI: Charlson comorbidity index
SMR: standardized morbidity ratio

## Background

Bladder cancer (BC) is among the 10 most common cancers globally [1], accounting for 573,000 new diagnoses annually [2]. The most significant clinical presentation is monosymptomatic macroscopic hematuria [3], justifying evaluation in Denmark [4]. Antithrombotic medication‐induced hematuria has been suggested to reduce diagnostic delay of undiagnosed BC (unmasking) [5].

Aspirin is an antiplatelet medication through cyclooxygenase (COX)‐1inhibition [6], however, only weakly associated with hematuria [7, 8]. Whether aspirin unmasks undiagnosed BCs is only sparsely addressed in the existing literature. A Canadian cohort study found that aspirin initiators received more urological procedures in a 12‐year follow‐up than individuals with no aspirin use [9]. A lower stage distribution at BC diagnosis could also indicate unmasking. Several studies comparing stage distribution at BC diagnosis have found a lower stage among users of any antithrombotic medication including aspirin compared with non‐users, however, without providing estimates for aspirin users only [10, 11, 12]. A small 2022 study provided antithrombotic medication subgroup estimates, but conversely to the priorly mentioned studies, found no difference in stage distribution among any of these, including the group of aspirin users [5].

Aspirin's role in the timeliness of BC diagnosis is important for the discussion on aspirin's association with BC incidence. Although BC prevention by aspirin would be expected, in line with aspirin's primary preventive abilities on other cancers [13, 14, 15], convincing associations between aspirin use and BC incidence are lacking in the literature [16, 17, 18, 19, 20, 21, 22]. If aspirin unmasks undiagnosed BCs, this may increase BC incidence among aspirin users compared with non‐users during short‐term follow‐up in studies.

Non‐aspirin, non‐selective, non‐steroidal anti‐inflammatory drugs (NSAIDs) are also COX‐1 inhibitors, although with less pronounced antiplatelet abilities than aspirin [7, 23]. Interestingly, NSAIDs are associated with a reduced incidence of BC in the literature [19], although the underlying mechanism is hypothesized to be similar to that of aspirin [6]. To our knowledge, no prior studies have addressed whether NSAID initiation unmasks undiagnosed BCs.

To describe cystoscopy incidence in the aspirin and NSAID initiating population as a proxy for altered diagnostic delay, we conducted this Danish nationwide study comparing aspirin and NSAID initiators with never‐users regarding: (1) cystoscopy rates, (2) BC prevalence at cystoscopy, and (3) prevalence of invasive stage at diagnosis.

## Methods

### Data sources

The Danish tax‐financed healthcare system provides residents with free healthcare [24]. The Danish medical registries hold near‐complete national individual level records and cross‐linkage is possible by a unique personal identifier [25]. The Danish Prescription Registry (prescription registry) [26] holds records of all redeemed medication prescriptions in Denmark since 1995 and provided exposure and comedication information. The Danish Civil Registration System [27] holds complete records of all living persons in Denmark since 1968 and provided covariate information. The Danish National Patient Registry (patient registry) [28] holds records of all inpatient admissions since 1977 and of all outpatient and emergency room visits since 1995 and provided outcome variable and covariate information. Although symptoms, such as hematuria, sometimes are registered, for instance as procedure codes when no other pathology is found, or as referral related codes, they are reported inconsistently and we refrained from using them. The Danish Pathology Registry (pathology registry) [29] holds records of all pathological specimens and diagnoses in Denmark since 1997 and provided outcome variable information. Definitions of codes used are provided in Table S1.

### Study populations

#### Cystoscopy assessment

To describe cystoscopy rates, we identified two separate index cohorts consisting of all first‐time aspirin or NSAID users aged 18 years or older, registered in the prescription registry between January 1, 2005 and December 31, 2023. For inclusion, we required at least two redeemed prescriptions for NSAIDs or low‐dose aspirin (pill dosage: <150 mg/day) or high‐dose aspirin (pill dosage: ≥150 mg/day), within 4 months, and set the index date to 4 months after first redemption. We required available lookback information from the start of the respective registries or date of birth, whichever was most recent, up to the index date. We excluded individuals without available pill dosage information.

We excluded individuals who, on the index date, had a prior record of cancer diagnoses (except non‐melanoma skin cancer), urological pTa tumors or carcinoma in situ, redeemed antithrombotic medication prescriptions, cystoscopy procedures, hematuria diagnoses, chronic bleeding conditions, or certain urological diseases requiring cystoscopy as part of the work‐up (neurogenic bladder disease, chronic interstitial cystitis, and incontinence).

We sampled a comparison cohort of unexposed individuals, representing the general population, for each index cohort using risk‐set sampling, sampled 1:10 with replacement [30], meaning that each unexposed individual was eligible to be matched with multiple exposed individuals. Matching was conducted by birth year, sex, and municipality of residence at the index date. Unexposed individuals had to fulfill the same inclusion‐ and exclusion criteria as the exposed individuals and additionally had to be NSAID, high‐ or low‐dose aspirin unexposed before the index date. We excluded individuals from the index cohorts without eligible matches.

#### Bladder cancer detection and invasive stage assessment

To describe BC prevalence at cystoscopy and the prevalence of invasive stage prevalence at diagnosis, we further identified all first‐time cystoscopy recipients (flexible or rigid) in Denmark (regardless of membership in the matched cohorts) between January 1, 2005 and December 31, 2022. Among these, we defined NSAID or aspirin initiators as individuals with at least two redeemed prescriptions for NSAIDs, low‐, or high‐dose aspirin, respectively, in the 12 months before cystoscopy with no redeemed prescriptions for any of these drugs during 1–10 years before cystoscopy. We further included never‐users, defined as individuals with no redemptions of these drugs in 10 years before cystoscopy. The same inclusion‐ and exclusion criteria as for the cystoscopy rate analysis were applied, except for any hematuria diagnosis prior to cystoscopy, as hematuria is one of the main cystoscopy referral criteria.

### Outcome

#### Cystoscopy assessment

We retrieved data on first‐time cystoscopy (flexible or rigid) in the patient registry [28]. In the public Danish healthcare system cystoscopies are always performed by physician referral.

#### Bladder cancer detection and invasive stage assessment

We defined BCs occurring within 1 year after the index cystoscopy as prevalent cases at the index cystoscopy; thereby acknowledging system delays during different steps of the BC diagnostic process. We obtained information on BC prevalence and stage using the validated algorithm by the Danish BC Database [31]. Thereby, only BC diagnoses with stage (strata: pTa‐tumor, carcinoma in situ or T1+ BCs) and time concomitance in the patient and pathology registry were regarded. Time concomitance was defined as a first‐time diagnosis in the patient registry within 90 days before to 365 days after a histopathological diagnosis in the pathology registry. We defined the date of BC diagnosis as the pathology registry's diagnosis date. If more than one diagnostic stage was recorded concurrently in both registries in, for example, patients with multiple tumors, only the worst stage at the first‐ever date of diagnosis was regarded.

### Covariates

In all analyses, we included the baseline covariates: age, sex, marital status (yes/no), and comorbidity. For comorbidity, we used the Charlson comorbidity index (CCI) score, based on all diagnoses recorded in the patient registry any time before the index date [32], categorized as a score of 0, 1–2, or 3+. Components of the CCI score, which were further included as individual variables into the weighting model, were included as yes/no variables. We included calendar year, as the indications for aspirin‐ and NSAID prescription changed during the study period [33, 34]. In the cystoscopy assessment, we further counted number of weeks with in‐house contact to a general practitioner in one to 5 years prior to the index date as a measure of health‐seeking behavior.

### Statistical analyses

#### Cystoscopy assessment

We followed individuals from the index date for up to 1 year or until the first occurrence of: cystoscopy, death, emigration, or initiation of any other antithrombotic medication than the index medication (index cohort) or any antithrombotic medication (comparison cohort). We estimated and plotted the cumulative incidence of cystoscopy using the Aalen‐Johansen estimator [35] to account for death as a competing risk. We reported the cumulative incidence proportion of cystoscopies during the 1‐year follow‐up. For relative comparisons, we computed cystoscopy hazard ratios by stratified COX regression allowing for different baseline hazards within each matched set with adjustment for CCI score. We used the robust sandwich estimator to create 95% confidence intervals (CIs). We used log‐minus‐log plots to assess the proportionality assumptions for the exposure and found them acceptable.

In sensitivity analyses, we repeated the primary analysis with (1) age restricted (below or above 70 years of age), (2) requirement of only one redeemed index drug prescription, setting the index date at first redemption, (3) a reduced follow‐up period of 3 months, and (4) analysis for each sex separately.

#### Bladder cancer detection and invasive stage assessment

We presented crude prevalences of non‐invasive (pTa and carcinoma in situ) and invasive (T1+) BC by aspirin or NSAID use. To account for baseline differences between exposure groups, we applied standardized morbidity ratio weighting (SMRW). For this, we used logistic regression to calculate propensity scores, which were used to compute weights in the unexposed cohort (propensity score/(1 − propensity score)), whereas the weight in the exposed cohort was always 1. The logistic regression included the same covariates as those adjusted for in the cystoscopy rate analysis, along with marital status, ischemic heart disease, peripheral vascular disease, cerebrovascular disease, congestive heart disease, diabetes mellitus, dementia, chronic pulmonary disease, and connective tissue disease, all as yes/no variables based on all available information prior to the index date. Weighting was conducted withhin age strata (70 years </+) whereafter the datasets were combined again. We plotted absolute mean differences and required less than 0.1 in difference (Fig. S2a–d). We used the robust sandwich estimator to create 95% CIs. For each analysis, we presented crude and weighted prevalence ratios with 95% CIs.

In sensitivity analyses, we repeated the primary analysis with (1) requirement of only one redeemed index drug prescription for exposed individuals, (2) the BC outcome defined solely by the patient registry, (3) requirement of no aspirin or NSAID prescription before 1 year before the index cystoscopy, (4) exclusion of prior users only based on the exposure defining index drug, (5) age restricted (below or above 70 years of age), and (6) analysis for each sex separately.

We used the statistical software packages R, v4.3.3 and SAS, v.9.4 (SAS Institute) for data management and analyses. The study was reported to the Danish Data Protection Agency through registration at Aarhus University (record number KEA‐2017‐36/812). In accordance with Danish regulations governing analysis of registry data, no Ethics Committee approval was required.

## Results

### Cystoscopy assessment

For the aspirin comparison, we included 50,771 aspirin initiators with 492,445 matched never‐users; 156,191 NSAID initiators and 1,549,155 matched never‐users for the NSAID comparison (Tables 1 and 2 and Table S2). High‐dose aspirin initiators accounted for 2201 aspirin initiators. Cumulative number of weeks with a visit to the general practitioner were slightly higher among aspirin (median 10; interquartile range, IQR: 5, 18) and NSAID (9; IQR: 4, 15) initiators than their respective comparison cohorts (7; IQR: 3, 13, and 6; IQR 3, 11).

**Table 1:** Baseline characteristics for the three study populations of aspirin initiators and never‐users.

	Cystoscopy assessment^a^		Bladder cancer detection assessment^b^			Invasive stage assessment^c^		
	Initiator	Never‐user	Initiator	Never‐user	SMD^d^	Initiator	Never‐user	SMD^d^
No.	50,771	492,445	683	59,912		75	4176	
Sex, male	25,989 (51)	251,965 (51)	474 (69)	38,228 (64)	0.005	52 (69)	3097 (74%)	0.015
Age								
Median (IQR)	67 (59, 76)	67 (59, 75)	70 (63, 78)	59 (44, 69)	0.009	71 (67, 78)	69 (62, 76)	0.006
<50 years	4826 (9.5)	48,388 (9.8)	81 (12)	11,176 (19)		<5 (<7.0)	572 (14)	
50–59 years	8226 (16)	81,921 (17)	27 (4.0)	19,813 (33)		<5 (<7.0)	209 (5.0)	
60–69 years	15,802 (31)	157,765 (32)	214 (31)	14,682 (25)		24 (32)	1343 (32)	
70–79 years	13,554 (27)	134,602 (27)	230 (34)	10,486 (18)		33 (44)	1429 (34%)	
80+ years	8363 (17%)	69,769 (14%)	131 (19%)	3755 (6.3%)		13 (17%)	623 (15%)	
Calendar year of the index date					0.009			0.025
2005–2009	28,600 (56)	279,011 (57)	289 (42)	14,204 (24)		27 (36)	907 (22)	
2010–2015	14,404 (28)	138,627 (28)	273 (40)	25,630 (43)		32 (43)	1772 (42)	
2016–2023	7767 (15)	74,807 (15)	121 (18)	20,078 (34)		16 (21)	1497 (36)	
Married^e^	42,611 (84)	413,917 (84)	404 (59)	33,955 (57)	0.004	49 (65)	2618 (63)	0.008
Charlson comorbidity index level					0.027			0.010
0	34,129 (67)	427,553 (87)	302 (44)	47,469 (79)		36 (48)	3092 (74)	
1–2	13,916 (27)	58,258 (12)	278 (41)	10,629 (18)		26 (35)	922 (22)	
3+	2726 (5.4)	6634 (1.3)	103 (15)	1814 (3.0)		13 (17)	162 (3.9)	
Ischemic heart disease	1199 (2.4)	1796 (0.4)	26 (3.8)	259 (0.4)	0.029	<5 (<7.0)	31 (0.7)	0.012
Peripheral heart disease	3976 (7.8)	4284 (0.9)	115 (17)	699 (1.2)	0.042	9 (12.0)	72 (1.7)	0.009
Cerebrovascular disease	3218 (6.3)	6831 (1.4)	67 (9.8)	1056 (1.8)	0.030	6 (8.0)	81 (1.9)	0.029
Congestive heart disease	2010 (4.0)	2574 (0.5)	37 (5.4)	340 (0.6)	0.008	<5 (<7.0)	47 (1.1)	0.005
Diabetes mellitus	3435 (6.8)	8168 (1.7)	89 (13)	1586 (2.6)	0.019	13 (17)	113 (2.7)	0.019
Diabetes mellitus with organ damage	1566 (3.1)	3029 (0.6)	49 (7.2)	626 (1.0)	0.014	9 (12)	48 (1.1)	0.010
Chronic pulmonary disease	2971 (5.9)	17,942 (3.6)	22 (3.2)	275 (0.5)	0.021	<5 (<7.0)	20 (0.5)	0.048
Connective tissue disease	992 (2.0)	7166 (1.5)	72 (11)	3694 (6.2)	0.005	7 (9.3)	290 (6.9)	0.010

*Note*: Unless otherwise noted: numbers (%).

Abbreviation: IQR, interquartile range.

^a^ The baseline characteristics of the study cohort of first‐time aspirin initiators during the study period from 2005 to 2023 (initiators) with age, sex, and residence of municipality matched comparisons from the background population (never users).

^b^ The baseline characteristics of the study population of first‐time cystoscopy recipients in 2005–2022, who initiated first‐time aspirin treatment within 1 year before the cystoscopy (initiators) or did not use aspirin or NSAID 10 years before the cystoscopy (never users).

^c^ The baseline characteristics of the study population of first‐time cystoscopy recipients in 2005–2022, where a bladder cancer was diagnosed at the cystoscopy, and who initiated aspirin treatment within 1 year before the cystoscopy (initiators) or did not use aspirin or NSAID 10 years before the cystoscopy (never users).

^d^ Absolute standardized mean difference after weighting between the exposed group and never users.

^e^ Married or in registered partnership.

**Table 2:** Baseline characteristics for the three study populations of NSAID initiators and never‐users.

	Cystoscopy assessment^a^		Bladder cancer detection assessment^b^			Invasive stage assessment^c^		
	Initiator	Never‐user	Initiator	Never‐user	SMD^d^	Initiator	Never‐user	SMD^d^
No.	156,191	1,549,155	1414	59,912		66	4176	
Sex, male	78,076 (50)	775,372 (50)	886 (63)	38,228 (64)	0.001	43 (65)	3097 (74)	0.004
Age								
Median (IQR)	45 (28, 60)	45 (28, 60)	60 (46, 70)	59 (44, 69)	<0.001	68 (62, 75)	69 (62, 76)	0.003
<50 years	89,503 (57)	894,268 (58)	253 (18)	11,176 (19)		<15 (<24)	572 (14)	
50–59 years	26,657 (17)	266,462 (17)	437 (31)	19,813 (33)		<5 (<8.0)	209 (5.0)	
60–69 years	21,494 (14)	214,268 (14)	364 (26)	14,682 (25)		28 (42)	1343 (32)	
70–79 years	13,019 (8.3)	128,850 (8.3)	273 (19)	10,486 (18)		17 (26)	1429 (34)	
80+ years	5518 (3.6)	45,307 (2.9)	87 (6.2)	3755 (6.3)		10 (15)	623 (15)	
Calendar year of the index date					<0.001			0.014
2005–2009	58,948 (38)	585,986 (38)	300 (21)	14,204 (24)		14 (21)	907 (22)	
2010–2015	53,804 (34)	533,367 (34)	686 (49)	25,630 (43)		38 (58)	1772 (42)	
2016–2023	43,439 (28)	429,802 (28)	428 (30)	20,078 (34)		14 (21)	1497 (36)	
Married^e^	90,462 (58)	880,785 (57)	782 (55)	33,955 (57)	0.048	46 (70)	2618 (63)	0.002
Charlson comorbidity index level					<0.001			0.003
0	141,961 (91)	1,444,971 (93)	1039 (74)	47,469 (79)		39 (59)	3092 (74)	
1–2	13,010 (8.3)	95,057 (6.1)	323 (23)	10,629 (18)		<24 (<38)	922 (22)	
3+	1220 (0.8)	9127 (0.6)	52 (3.7)	1814 (3.0)		<5 (<8.0)	162 (3.9)	
Ischemic heart disease	208 (0.1)	1694 (0.1)	<5 (<0.4)	259 (0.4)	<0.001	<5 (<8.0)	31 (0.7)	<0.001
Peripheral heart disease	726 (0.5)	4996 (0.3)	29 (2.1)	699 (1.2)	<0.001	<5 (<8.0)	72 (1.7)	0.004
Cerebrovascular disease	1019 (0.7)	8122 (0.5)	29 (2.1)	1056 (1.8)	<0.001	<5 (<8.0)	81 (1.9)	0.020
Congestive heart disease	316 (0.2)	2402 (0.2)	14 (1.0)	340 (0.6)	<0.001	<5 (<8.0)	47 (1.1)	0.003
Diabetes mellitus	1859 (1.2)	13,125 (0.8)	55 (3.9)	1586 (2.6)	<0.001	<5 (<8.0)	113 (2.7)	0.006
Diabetes mellitus with organ damage	635 (0.4)	4516 (0.3)	16 (1.1)	626 (1.0)	<0.001	<5 (<8.0)	48 (1.1)	<0.001
Chronic pulmonary disease	5611 (3.6)	43,027 (2.8)	<5 (<0.4)	275 (0.5)	<0.001	<5 (<8.0)	20 (0.5)	0.006
Connective tissue disease	2458 (1.6)	10,517 (0.7)	111 (7.9)	3694 (6.2)	0.001	10 (15)	290 (6.9)	0.004

*Note*: Unless otherwise noted: numbers (%).

Abbreviations: IQR, interquartile range; NSAID, non‐aspirin, non‐steroidal, anti‐inflammatory drugs.

^a^ The baseline characteristics of the study cohort of first‐time NSAID initiators during the study period from 2005 to 2023 (initiators) with age, sex, and residence of municipality matched comparisons from the background population (never users).

^b^ The baseline characteristics of the study population of first‐time cystoscopy recipients in 2005–2022, who initiated first‐time NSAID treatment within 1 year before the cystoscopy (initiators) or did not use aspirin or NSAID 10 years before the cystoscopy (never users).

^c^ The baseline characteristics of the study population of first‐time cystoscopy recipients in 2005–2022, where a bladder cancer was diagnosed at the cystoscopy, and who initiated NSAID treatment within 1 year before the cystoscopy (initiators) or did not use aspirin or NSAID 10 years before the cystoscopy (never users).

^d^ Absolute standardized mean difference after weighting between the exposed group and never users.

^e^ Married or in registered partnership.

The crude incidence of cystoscopy during the 1‐year follow‐up was higher for aspirin and NSAID initiators compared with the comparison cohorts (Fig. S1). The unadjusted and adjusted cystoscopy hazard ratios were 1.66 (95% CI: 1.50–1.85) and 1.54 (95% CI: 1.38–1.72) in the aspirin comparison; 1.57 (95% CI: 1.45–1.71) and 1.56 (95% CI: 1.43–1.70) in the NSAID comparison (Table 3). We obtained similar results, when sex or age restricting, requiring only one redeemed prescription, reducing the follow‐up period to 3 months, and including low‐dose aspirin initiators only (Fig. S3a).

**Table 3:** Cystoscopy rates among first‐time aspirin or non‐steroidal anti‐inflammatory drug (NSAID) initiators in 2005–2023 and never‐using comparisons.

	Aspirin comparison		NSAID comparison	
	Initiator	Never‐user	Initiator	Never‐user
Total person‐time, days	46,385	466,849	151,179	1,509,659
Follow‐up end, no. (%)				
Full‐follow‐up of 1 year	42,685 (84)	441,684 (90)	146,324 (94)	1,469,347 (95)
Cystoscopy outcome	402 (0.8)	2435 (0.5)	622 (0.4)	3943 (0.3)
Death	1456 (2.9)	7788 (1.6)	1159 (0.7)	7507 (0.5)
Emigration	19 (<0.1)	228 (<0.1)	355 (0.2)	4313 (0.3)
Study‐end reached	815 (1.6)	8030 (1.6)	4379 (2.8)	43,608 (2.8)
Opposing index drug initiated	3048 (6.0)	24,330 (4.9)	1843 (1.2)	11,286 (0.7)
Other antithrombotic medication initiated	2346 (4.6)	7950 (1.6)	1509 (1.0)	9151 (0.6)
Cumulative incidence^a^ (95% CI)	0.85 (0.77–0.93)	0.52 (0.5–0.54)	0.41 (0.38–0.44)	0.26 (0.25–0.27)
Hazard ratio (95% CI)				
Crude	1.66 (1.50–1.85)	Reference	1.57 (1.45–1.71)	Reference
Adjusted	1.54 (1.38–1.72)	Reference	1.56 (1.43–1.70)	Reference

Abbreviations: CI, confidence interval; IQR, interquartile range; no., number.

^a^ Crude bladder cancer cumulative incidence proportion at 1 year follow‐up.

### Bladder cancer detection and invasive stage assessment

Among 287,181 eligible first‐time cystoscopy recipients, we included 683 first‐time aspirin initiators, 1414 first‐time NSAID initiators, and 59,912 never‐users (Tables 1 and 2 and Table S3). High‐dose aspirin initiators accounted for 30 aspirin initiators.

The crude BC prevalence was higher among aspirin initiators (11%) compared with never‐users (7%), whereas weighted analysis, controlling for differences at time of cystoscopy, showed similar prevalences (SMRW prevalence ratio: 0.97 [95% CI, 0.76–1.22], Table 4). The crude BC prevalence was lower among NSAID initiators (5%) compared with never‐users (7%), which was consistent in weighted comparison (SMRW prevalence ratio: 0.65 [95% CI, 0.51–0.82]). Among aspirin initiators, those who were female or younger than 70 years had a higher proportion of BCs detected at cystoscopy compared with the overall analysis.

**Table 4:** Bladder cancer‐ and stage prevalence at first‐time cystoscopy among exposure groups.

	Aspirin initiator	NSAID initiator	Never‐user
**Bladder cancers, no (%)**	75 (11)	66 (5)	4176 (7)
Non‐invasive bladder cancers, no (%)	40 (53)	30 (45)	2085 (50)
Invasive bladder cancers, no (%)	35 (47)	36 (55)	2091 (50)
**Bladder cancer prevalence ratio (95% CI)**			
Crude	1.58 (1.26–1.94)	0.67 (0.52–0.84)	Reference
SMR weighted	0.97 (0.76–1.22)	0.65 (0.51–0.82)	Reference
**Invasive stage prevalence ratio (95% CI)**			
Crude	0.93 (0.71–1.16)	1.09 (0.85–1.33)	Reference
SMR weighted	0.81 (0.63–1.03)	1.07 (0.85–1.34)	Reference

Abbreviations: CI, confidence interval; no., number; SMR, standardized morbidity ratio.

The crude prevalence of invasive stage at BC diagnosis was slightly lower among aspirin initiators (47%) compared with never‐users (50%). This difference was more pronounced in the weighted comparison (SMRW prevalence ratio: 0.81 [95% CI, 0.63–1.03]). The prevalence of invasive stage was similar among NSAID initiators (55%) and never‐users (50%, SMRW prevalence ratio: 1.07 [95% CI, 0.85–1.34]). In the weighted analysis, female aspirin initiators compared with female never‐users showed the largest difference in stage at diagnosis with a prevalence ratio of 0.51 (95% CI, 0.29–0.91, Fig. S3b,c). We obtained similar results, when age restricting, requiring only one redeemed prescription, using only the patient registry as our outcome source, excluding based on all available lookback, and excluding based on prior prescription of the index drug only (Fig. S2b,c).

## Discussion

In this population‐based study, aspirin initiators received more cystoscopies than never‐users from the background population. Upon cystoscopy, recent aspirin initiators had similar BC prevalence, but a lower prevalence of invasive stage compared with never‐users. Our interpretation is that individuals initiating aspirin treatment represent a patient population with an a priori higher incidence of bladders cancer and that their higher cystoscopy rate reflect this and thus is clinically relevant. The combination of a larger proportion of relevant cystoscopies and a lower prevalence of invasive stage at diagnosis may represent the unmasking of otherwise asymptomatic BCs. This pattern was more pronounced in analysis restricted to female individuals, suggesting that the unmasking of previously undiagnosed BCs may be more pronounced among female individuals. We further found that NSAID initiators received more cystoscopies than never‐users. However, NSAID initiators had a lower BC prevalence at cystoscopy and similar stage distribution as never‐users. This suggests that the higher cystoscopy rate may not have been clinically warranted.

Our findings support the findings in the Canadian cohort study, which found that aspirin initiators received more urological procedures compared with unexposed individuals [9]. Although the Canadian study further found increased BC risks 6 months after the hematuria‐related complication for all antithrombotic agents combined, we can add to the existing knowledge that aspirin initiators also have increased BC prevalence, when considering the combination of more cystoscopies and similar BC prevalences upon cystoscopy than the general population. To our knowledge, only one existing study has addressed BC prevalences for aspirin users, finding a prevalence of 22% among aspirin users, which were evaluated for macroscopic hematuria [8]. Not restricting our analysis to hematuria patients only may explain the slightly lower BC prevalence in our aspirin cohort.

Our findings align with three analyses that compared BC stage and grade at diagnosis for antithrombotic medication users and non‐users [10, 11, 12]. Despite no stratification by antithrombotic medication class, all reported lower stages and grades of BC among antithrombotic medication users. In contrast, another analysis from 2022, which stratified by antithrombotic medications classes, concluded that there was no association between users of any antithrombotic medications and BC stage [5]. We only included new users of aspirin, which unlike prevalent users may represent a population, where differences are more pronounced, as we would expect side‐effect vulnerable patients to be non‐continuers of the treatment and thus possibly not be represented in a prevalent user cohort. Further, we defined invasive stage based on connective tissue invasion or worse corresponding to all stages from T1 to direct extravesical tumor growth.

A lower stage at diagnosis after aspirin exposure could also reflect a potential protective effect of aspirin on tumor growth or progression. However, because the exposed individuals in our study were on average exposed to aspirin for less than 1 year before diagnosis, such a potential protective effect is a less likely the explanation for the observed lower stage at diagnosis.

We are not familiar with any studies addressing NSAID initiation and its association with cystoscopy rates, BC prevalence at cystoscopy or invasive stage prevalence at BC diagnosis. The NSAID initiating cohort was young in comparison with the overall median age of 75 years in Danish BC patients [36]. However, the associations were similar, when we restricted to individuals above 70 years. We considered whether prescription NSAID use could be a marker of poorer health, particularly among the younger individuals in our study population. Although both aspirin and NSAIDs have antiplatelet effects, the effect is not as profound for NSAIDs than for aspirin [37]. Therefore, we interpret our findings as if the underlying mechanisms for the increased cystoscopy rates among NSAID initiators were unlikely to be related to hematuria complications. Although aspirin is often given as a treatment for a known condition, NSAIDs are also prescribed for symptoms, where the underlying reason not always is diagnosed at the time of prescription.

Sex disparities in BC diagnostics are previously extensively described in the literature, with female BC patients having higher stage at diagnosis [38]. Our findings further strengthened the assumption that diagnostic delay may be more pronounced among female individuals than male individuals.

### Strengths/limitations

This was a large population‐based study with near‐complete follow‐up reflecting routine clinical care. There are some limitations to be acknowledged. First, we observed more comorbidity among aspirin initiators than never users. Aspirin is used for secondary cardiovascular disease prevention [33], and cardiovascular diseases share several risk factors with BC such as smoking [40, 41], which may have influenced the associations both toward and away from an association. Second, aspirin and NSAID medications were also available as over‐the‐counter medications. Thus, we cannot rule out some exposure misclassification, which may have biased our relative estimates toward no association. However, the problem of over‐the‐counter use in observational studies of prescriptions‐based use has been addressed previously based on the Danish Prescription Database, where it, based on sales statistics for NSAIDs, was found that the prevalence of true use was misclassified as non‐use peaked at 4.7% in 2012 for NSAID, whereas the misclassification of true low‐dose aspirin use as non‐use has remained around 1% since 2005, and it was concluded that the influence of non‐recorded aspirin or NSAID use was virtually negligible [39]. Third, relying on registries, we lacked information on some covariates of interest, such as smoking habits, whereas other covariates, such as a prior hematuria diagnosis, as previously mentioned were assumed to be inconsistently reported and refrained from being used. If they were reliably reported, they could have added further clarification on the associations. Although hematuria‐driven unmasking was the basis for our hypothesis, differences in factors, such as health‐seeking behaviors, may also exist between initiators and never‐users. We consider that shorter diagnostic delay for any underlying reason related to aspirin or NSAID initiation is valuable information in the interpretation of aspirin and NSAIDs association with BC incidence. Finally, diagnostic delay may be different in other settings depending on referral guidelines and availability of cystoscopy, which in Denmark requires physician referral.

### Implications

The results of this study may be highly relevant in the understanding of aspirins and NSAIDs association with BC incidence, as observational studies may need to take differences in diagnostic timeliness into account in the aspirin‐using population.

## Conclusion

Aspirin and NSAID initiators received more cystoscopies than the background population within 1 year of initiation. At cystoscopy, aspirin initiators had similar BC prevalences, but a lower stage distribution at diagnosis than never‐users, which may reflect unmasking of asymptomatic BCs. Contrary, we found no indication of unmasking asymptomatic BCs in NSAID initiators.

## Author contributions

**Malene Söth Hansen**: Investigation; conceptualization; writing—original draft; methodology; validation; visualization; writing—review and editing; software; formal analysis; project administration; data curation; resources. **Lars Dyrskjøt**: Writing—review and editing; supervision; methodology. **Uffe Heide‐Jørgensen**: Conceptualization; methodology; visualization; writing—review and editing; supervision. **Mette Nørgaard**: Investigation; conceptualization; funding acquisition; methodology; validation; visualization; writing—review and editing; software; formal analysis; project administration; data curation; supervision; resources. **Jørgen Bjerggaard Jensen**: Writing—review and editing; supervision; methodology.

## Conflict of interest statement

Malene Söth Hansen, Uffe Heide‐Jørgensen, Jørgen Bjerggaard Jensen, and Mette Nørgaard have nothing to disclose. Lars Dyrskjøt has sponsored research agreements with C2i Genomics, Veracyte, Natera, AstraZeneca, Photocure, and Ferring and has an advisory/consulting role at Ferring, MSD, Cystotech, AstraZeneca and UroGen. Lars Dyrskjøt has received speaker honoraria from AstraZeneca, Pfizer, and Roche and travel support from MSD. The Department of Clinical Epidemiology is involved in studies with funding from various companies as research grants to (and administered by) Aarhus University, Denmark.

## Funding information

Danish Cancer Society [Grant number: R321‐A17497]. The funding sources did not contribute to any steps of this work, such as study design, collection, analysis, and interpretation of data, writing of the report and decision to submit the article for publication.

## Supporting information




**Supplement Table S1**: Codes used in the study.
**Supplement Table S2**: Numbers of eligible, excluded and included individuals for cystoscopy rate assessment.
**Suplementary Figure 1A**: Cystoscopies conducted in one‐year follow‐up among aspirin initiators and never‐using comparisons.
**Suplementary Figure 1B**: Cystoscopies conducted in one‐year follow‐up among NSAID initiators and never‐using comparisons.
**Supplementary Figure 2A**: Absolute mean differences in the bladder cancer prevalence analysis before and after standardized morbidity weighting between aspirin initiators and never‐users.
**Supplementary Figure 2B**: Absolute mean differences in the bladder cancer prevalence analysis before and after standardized morbidity weighting between NSAID initiators and never‐users.
**Supplementary Figure 2C**: Absolute mean differences in the invasive stage prevalence analysis before and after standardized morbidity weighting between aspirin initiators and never‐users.
**Supplementary Figure 2D**: Absolute mean differences in the invasive stage prevalence analysis before and after standardized morbidity weighting between NSAID initiators and never‐users.
**Supplement Figure S3A**: forest plot of sensitivity analyses in the cystoscopy rate assessment.
**Supplement Figure S3B**: forest plot of sensitivity analyses in the bladder cancer detection assessment.
**Supplement Figure S3C**: forest plot of sensitivity analyses in the invasive stage assessment.
