# Supplementary material for: Aspirin or non‐steroidal anti‐inflammatory drug initiation and subsequent bladder cancer evaluation
Source: J Intern Med. 2026 Jun 1;300(2):193–203. doi: 10.1111/joim.70115 (PMC13327425; doi:10.1111/joim.70115)
Supplement: Supplementary file 1 — Supplement Table S1: Codes used in the study. Supplement Table S2: Numbers of eligible, excluded and included individuals for cystoscopy rate assessment. Suplementary Figure 1A: Cystoscopies conducted in one‐year follow‐up among aspirin initiators and never‐using comparisons. Suplementary Figure 1B: Cystoscopies conducted in one‐year follow‐up among NSAID initiators and never‐using comparisons. Supplementary Figure 2A: Absolute mean differences in the bladder cancer prevalence analysis before and after standardized morbidity weighting between aspirin initiators and never‐users. Supplementary Figure 2B: Absolute mean differences in the bladder cancer prevalence analysis before and after standardized morbidity weighting between NSAID initiators and never‐users. Supplementary Figure 2C: Absolute mean differences in the invasive stage prevalence analysis before and after standardized morbidity weighting between aspirin initiators and never‐users. Supplementary Figure 2D: Absolute mean differences in the invasive stage prevalence analysis before and after standardized morbidity weighting between NSAID initiators and never‐users. Supplement Figure S3A: forest plot of sensitivity analyses in the cystoscopy rate assessment. Supplement Figure S3B: forest plot of sensitivity analyses in the bladder cancer detection assessment. Supplement Figure S3C: forest plot of sensitivity analyses in the invasive stage assessment. [file JOIM-300-193-s001.docx]

| Supplement table S1: Codes used in the study | | | |
| --- | --- | --- | --- |
| Exposure definition |  | | |
| Aspirin |  | | |
| Low-dosis | ATC: N02BA01^a^, B01AC06 | | |
| High-dosis | ATC: N02BA01^a^, N02BA51, N02AJ07 | | |
| Non-selective NSAID | ATC: M01AE01, M01AE02, M01AE03, M01AE14, M01AC01, M01AC02, M01AG02 | | |
| Outcome definition – cystoscopy rates | | | |
| Cystoscopy | Procedure codes: KUKC02, KUKC05 | | |
| Inclusion definition – detection rates | | | |
| Cystoscopy | | Procedure codes: KUKC02, KUKC05 | |
| Outcome definition – detection rates  *Bladder cancer outcome definition from the Danish National Patient Registry* | | | |
| Ta-tumors | ICD-10: D303, D095 | | |
| Carcinoma in situ | ICD-10: D090 | | |
| Invasive bladder cancers | ICD-10: C679 | | |
| *Bladder cancer outcome definition from the Danish National Pathology Registry* | | | |
| Ta-tumors | SNOMED: T74*** & M81301, T74*** & M81302, ÆF181A | | |
| Carcinoma in situ | SNOMED: T74*** & M81202, ÆF1810 | | |
| Invasive bladder cancers | SNOMED: T74*** & M80**3-M84**3 | | |
| Exclusion variable definition | | | |
| Status codes in the Danish Civil Registration System indicating possible missing lookback periods or not Danish recidency | | Status-codes**^b^**: 05, 07, 20, 30, 50, 60, 70, 80 or 90 | |
| All cancers | | ICD-10: C00-C43, C45-C96, C99, | |
| pTa and carcinoma in situ | | ICD-10: D090, D095, D303 | |
| Hematuria | | ICD-10: R31, N02 | |
| Bleeding disorders  (not medically induced) | | ICD-10: D66, D67, D680, D681, D682, D684, D693, D469, I780, I788A | |
| Urological disease with routine cystoscopy in work-up | | ICD-10: N310, N301, N393, N394 | |
| Censuring variables | |  | |
| Non-aspirin antiplatelet drugs | | ATC: B01AC01-B01AC05, B01AC07-B01AC11, B01AC13, B01AC15-B01AC19, B01AC21-B01AC27, B01AC30 | |
| Anticoagulants | | ATC: B01AA, B01AB, B01AE, B01AF, B01AX | |
| Charlson Comorbidity Index components | | | **Score** |
| Myocardial infarction | ICD-08: 410  ICD-10: I21; I22; I23 | | 1 |
| Congestive heart failure | ICD-08: 427.09; 427.10; 427.11; 427.19; 428.99; 782.49  ICD-10: I50; I11.0; I13.0; I13.2 | | 1 |
| Peripheral vascular disease | ICD-08: 440; 441; 442; 443; 444; 445  ICD-10: I70; I71; I72; I73; I74; I77 | | 1 |
| Cerebrovascular disease | ICD-08: 430-438  ICD-10: I60-I69; G45; G46 | | 1 |
| Dementia | ICD-08: 290.09-290.19; 293.09  ICD-10: F00-F03; F05.1; G30 | | 1 |
| Chronic pulmonary disease | ICD-08: 490-493; 515-518  ICD-10: J40-J47; J60-J67; J68.4; J70.1; J70.3; J84.1; J92.0; J96.1; J98.2; J98.3 | | 1 |
| Connective tissue disease | ICD-08: 712; 716; 734; 446; 135.99  ICD-10: M05; M06; M08; M09; M30; M31; M32; M33; M34; M35; M36; D86 | | 1 |
| Ulcer disease | ICD-08: 530.91; 530.98; 531-534  ICD-10: K22.1; K25-K28 | | 1 |
| Mild liver disease | ICD-08: 571; 573.01; 573.04  ICD-10: B18; K70.0-K70.3; K70.9; K71; K73; K74; K76.0 | | 1 |
| Diabetes type1  Diabetes type2 | ICD-08: 249.00; 249.06; 249.07; 249.09  ICD-10: E10.0, E10.1;  ICD-08: 250.00; 250.06; 250.07; 250.09  ICD-10: E10.9; E11.0; E11.1; E11.9 | | 1 |
| Hemiplegia | ICD-08: 344  ICD-10: G81; G82 | | 2 |
| Moderate to severe renal disease | ICD-08: 403; 404; 580-583; 584; 590.09; 593.19; 753.10-753.19; 792  ICD-10: I12; I13; N00-N05; N07; N11; N14; N17-N19; Q61 | | 2 |
| Diabetes with end organ damage | ICD-08: 249.01-249.05; 249.08  ICD-10: E10.2-E10.8  ICD-08: 250.01-250.05; 250.08  ICD-10: E11.2-E11.8 | | 2 |
| Any tumor | ICD-08: 140-194  ICD-10: C00-C75 | | 2 |
| Leukemia | ICD-08: 204-207  ICD-10: C91-C95 | | 2 |
| Lymphoma | ICD-08: 200-203; 275.59  ICD-10: C81-C85; C88; C90; C96 | | 2 |
| Moderate to severe liver disease | ICD-08: 070.00; 070.02; 070.04; 070.06; 070.08; 573.00; 456.00-456.09  ICD-10: B15.0; B16.0; B16.2; B19.0; K70.4; K72; K76.6; I85 | | 3 |
| Metastatic solid tumor | ICD-08: 195-198; 199  ICD-10: C76-C80 | | 6 |
| AIDS | ICD-08: 079.83  ICD-10: B21-B24 | | 6 |
| General practitioner visits | V_KONTAKT==80, C_YDELSESNR == 101 | |  |
| Abbreviations: ICD = International Classification of Diseases, ATC= Anatomical Therapeutic Chemical.  ^a^ further subdivided into low- and high-dosis using vare-numbers  ^b^ Further information on status-codes descriptions from the civil registration system, please view: https://www.esundhed.dk/Dokumentation | | | |

| **Supplement table S2:** Numbers of eligible, excluded and included individuals for cystoscopy rate assessment | | | |
| --- | --- | --- | --- |
| **Variable** | **Aspirin** | | **NSAID** |
| Eligible | 97,484 | | 180,385 |
| Hematuria diagnosis prior to the index date | 3,229 | | 3,307 |
| Cancer diagnosis prior to the index date | 9,855 | 11,728 | |
| Urological- or bleeding disease* | 1,242 | 1,936 | |
| Cystoscopy prior to the index date* | 2,636 | 2,869 | |
| Antithrombotic medication prescription | 29,538 | 4,184 | |
| Eligible for matching | 50,984 | 156,361 | |
| No match found | 213 | 170 | |
| Matched initiators | 50,771 | 156,191 | |
| Matched comparisons | 492,445 | 1,549,155 | |

| **Supplement table S3:** Numbers of eligible, excluded and included individuals for bladder cancer detection assessment | | | |
| --- | --- | --- | --- |
| **Variable** | **Aspirin initiator** | **NSAID initiator** | **Non-initiators** |
| Eligible for group inclusion | 1,470 | 2,017 | 81,062 |
| Cancer prior to cystoscopy | 238 | 394 | 11,136 |
| Urological- or bleeding disease* | 38 | 79 | 3,203 |
| Antithrombotic medication prior to cystoscopy | 511 | 130 | 6,811 |
| Eligible for bladder cancer detection assessment | 683 | 1,414 | 59,912 |
| Elibible for invasive stage prevalence assessment | 75 | 66 | 4,176 |

**Suplementary figure 1A:** Cystoscopies conducted in one-year follow-up among aspirin initiators and never-using comparisons

**Suplementary figure 1B:** Cystoscopies conducted in one-year follow-up among NSAID initiators and never-using comparisons

| **Supplementary figure 2A:** Absolute mean differences in the bladder cancer prevalence analysis before and after standardized morbidity weighting between aspirin initiators and never-users |
| --- |
| 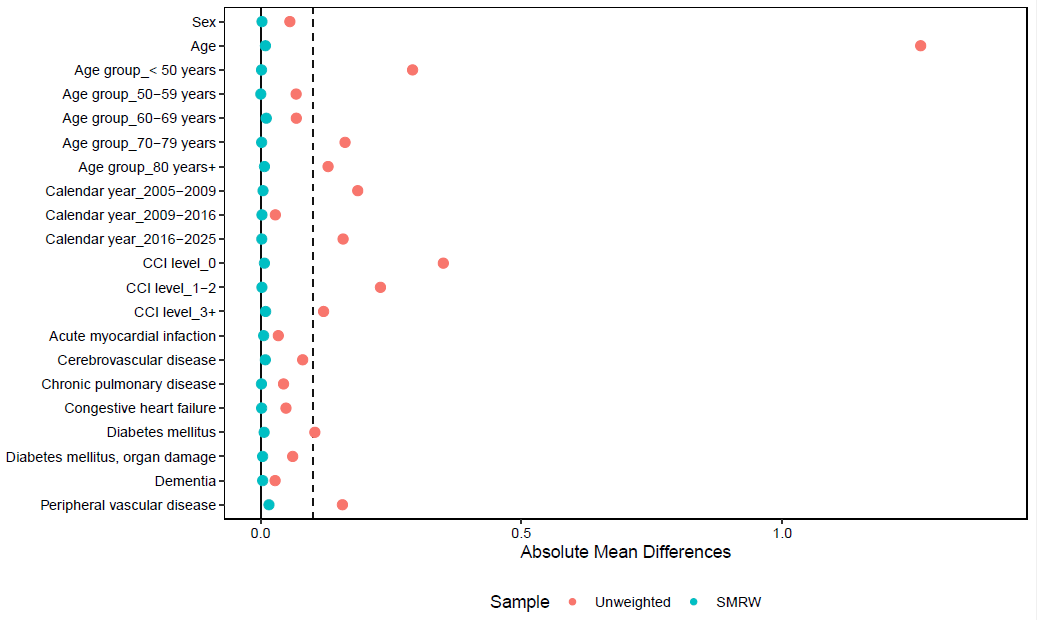 |
| Abbreviations: CCI = Charlson comorbidity index, SMRW = standardized morbidity ratio weighted |

| **Supplementary figure 2B:** Absolute mean differences in the bladder cancer prevalence analysis before and after standardized morbidity weighting between NSAID initiators and never-users |
| --- |
|  |


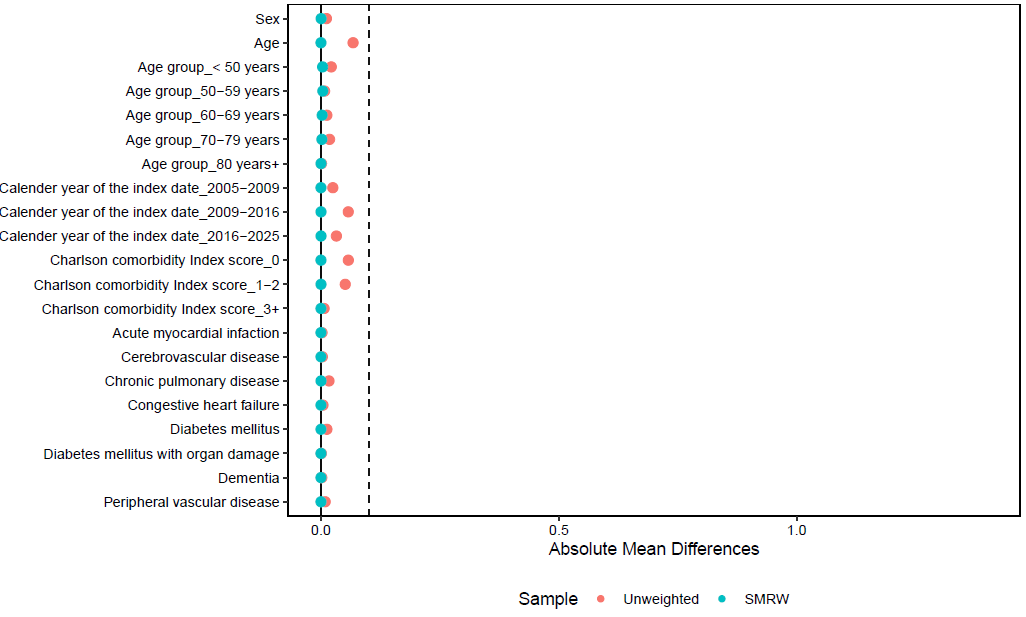


Abbreviations: CCI = Charlson comorbidity index, SMRW = standardized morbidity ratio weighted

| **Supplementary figure 2C:** Absolute mean differences in the invasive stage prevalence analysis before and after standardized morbidity weighting between aspirin initiators and never-users |
| --- |
|  |


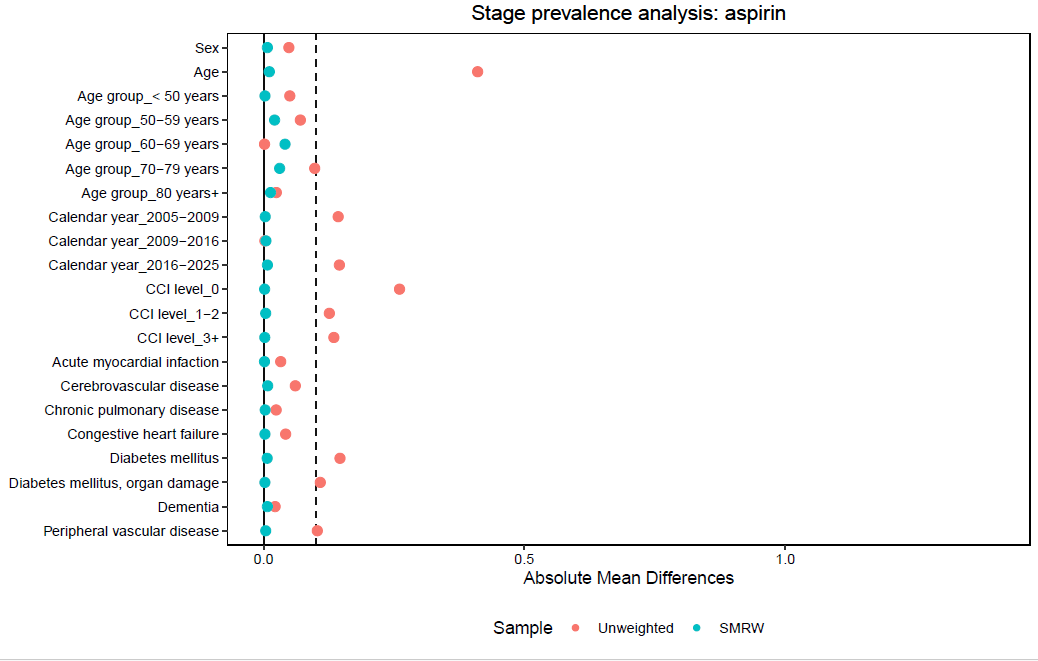


Abbreviations: CCI = Charlson comorbidity index, SMRW = standardized morbidity ratio weighted

| **Supplementary figure 2D:** Absolute mean differences in the invasive stage prevalence analysis before and after standardized morbidity weighting between NSAID initiators and never-users |
| --- |
|  |


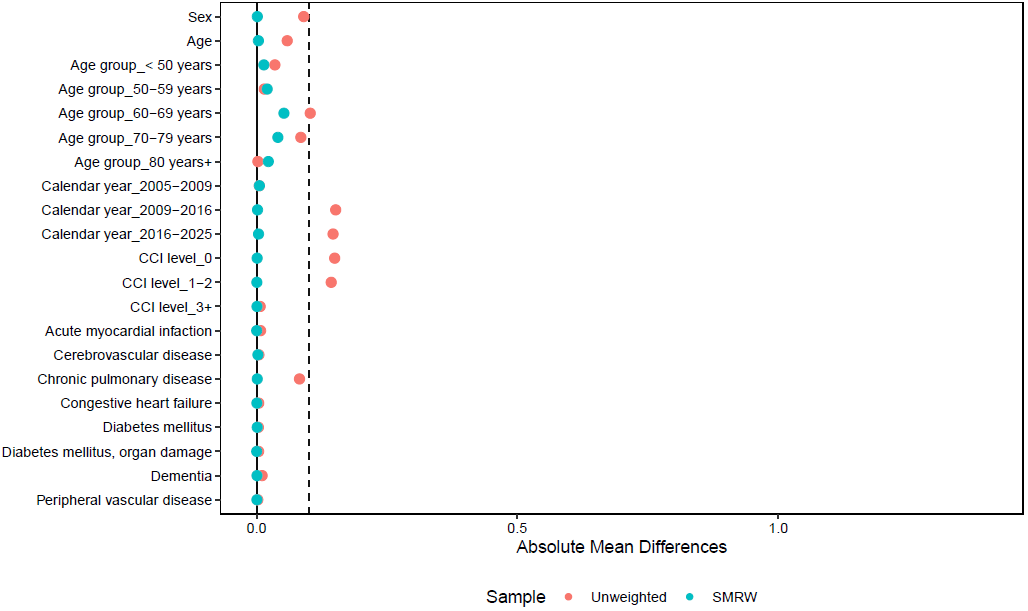


Abbreviations: CCI = Charlson comorbidity index, SMRW = standardized morbidity ratio weighted

|  |
| --- |
| **Supplement figure S3A:** forest plot of sensitivity analyses in the cystoscopy rate assessment  *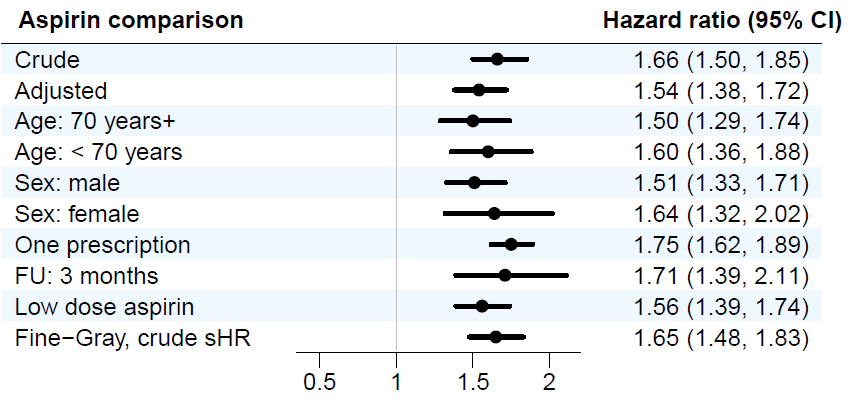* |
| *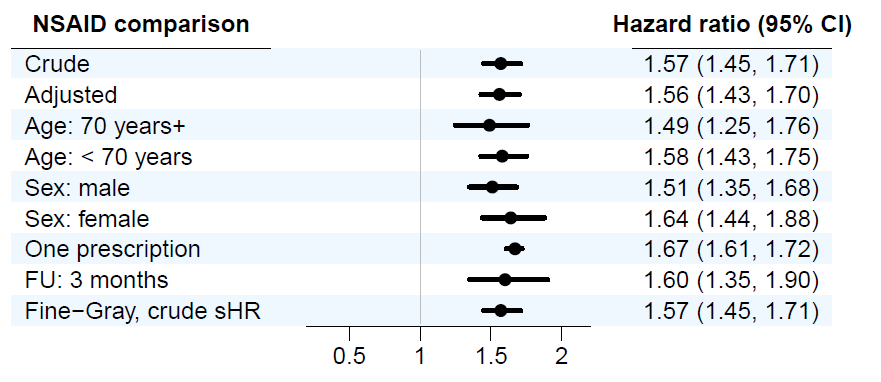* |
|  |
|  |
|  |
| Abbreviations: *One prescription:* the exposure is defined by one prescription only (conversely to two prescriotions in four months as in the main analysis). *FU: 3 months:* follow-up is ended at three months. *Low dose aspirin:* Only initiation of low dose aspirin is regarded.  All analyses except the “crude” analysis are adjusted for age, sex, Charlson comorbitidy index level and calendar year. |

| **Supplement figure S3B:** forest plot of sensitivity analyses in the bladder cancer detection assessment |
| --- |
| 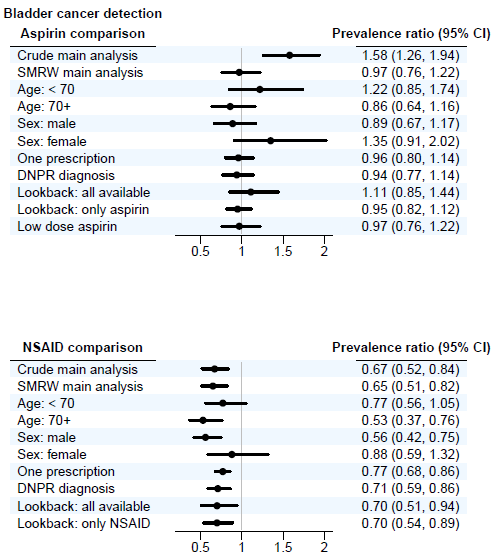 |
| Abbreviations: *SMRW main analysis* = standardized morbidity ratio weighted main analysis. *One prescription*: the exposure is defined by one prescription only (conversely to two prescriotions in four months as in the main analysis). *DNPR diagnosis*: The outcome variable only defined by the Danish National Patient Registry. *All lookback*: All available lookback applied from the latest of: date of birth or start of the Danish National Precription Registry for exclusion of previous users. *Lookback: only aspirin/Lookback: only NSAID:* Only the index drug in question is evaluated for exclusion of previous users. *Low dose aspirin*: Only initiation of low dose aspirin is regarded.  All analyses except the crude analyses are SMR weighted. |

| **Supplement figure S3C:** forest plot of sensitivity analyses in the invasive stage assessment |
| --- |
| **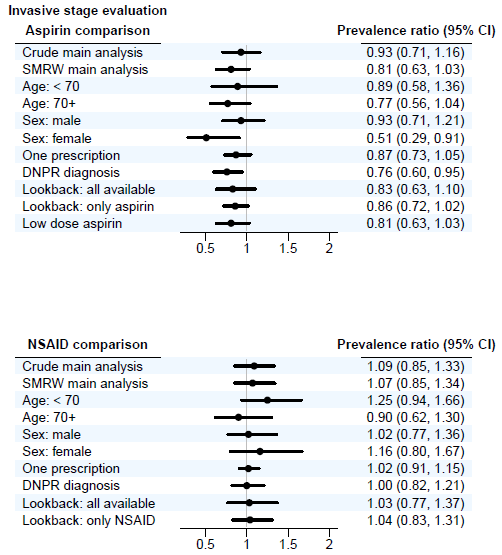** |
| Abbreviations: As above in figure S1B. All analyses except the crude analyses are SMR weighted. |
